# Leveraging artificial intelligence for livestock genetics and breeding

**DOI:** 10.1186/s40104-026-01527-3

**Published:** 2026-11-08

**Authors:** Di Zhu, Abbygail Wells, Rasmus Bak Stephansen, Hao Cheng, Jan Lassen, Georgios Banos, Mike Coffey, Lingzhao Fang, Bingjie Li

**Affiliations:** 1 Center for Quantitative Genetics and Genomics (QGG), Aarhus University, 8000 Aarhus, Denmark; 2 Animal Breeding & Genomics, Scotland’s Rural College (SRUC), Edinburgh, EH9 3JG UK; 3 The Royal (Dick) School of Veterinary Studies, University of Edinburgh, Easter Bush, Midlothian, EH25 9RG UK; 4 Department of Animal Science, University of California, Davis, Davis, CA 95616 USA; 5 VikingGenetics, 8960 Randers, Denmark

**Keywords:** Artificial intelligence, Complex-trait biology, Genomic prediction, Livestock genetics and breeding, Multi-omics, Phenotyping

## Abstract

The rapid development of artificial intelligence (AI) is transforming modern society including animal genetics and breeding which is essential for meeting global nutritional needs in supplying milk, meat, and eggs among the most complete sources of protein and micronutrients. Although animal breeding has a long history of using data-driven prediction methods, the application of modern AI approaches remains at a relatively early stage and shows substantial potential for wide-ranging applications in animal genetics and breeding. This study aims to review the current use of AI tools in livestock species across three major applications of high interest: AI-assisted phenotyping, AI-enabled dissection of complex-trait biology (focusing on AI-assisted GWAS and AI-for-omics), and AI-assisted genomic prediction. In addition, we discussed emerging methods and tools developed in human genetics that are not yet widely adopted in farm animals but may offer translational potential for future livestock studies. Looking ahead, AI breakthroughs in livestock genetics and breeding are likely to be driven by increases in data scale and resolution, enhanced computational capacities, standardized data infrastructure and benchmarking, and systematic method development and validation. From a livestock genetic improvement perspective, the key value of AI will lie in integrating AI-generated inputs, including improved phenotyping and biological insights, into practical breeding decisions through statistical modeling.

## Introduction

**Fig. 1:**
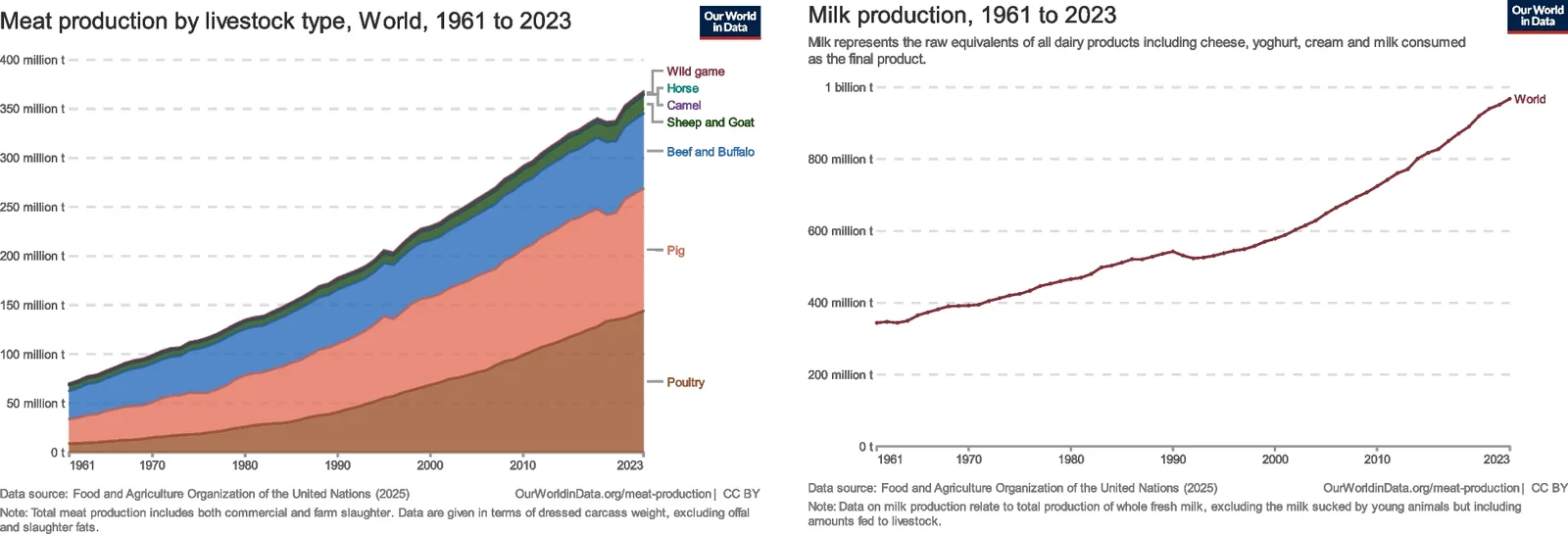
World meat production (left) and milk production (right) continue to rise to meet the nutritional needs of the world’s growing population [3]

The World Health Organisation estimates that over 700 million people globally are facing hunger, including 122 million who have been pushed into hunger between 2019 and 2023 [1]. The livestock industry is key to meeting the nutritional needs of the world population, with milk, meat and eggs providing some of the most complete sources of protein and micro-nutrients (Fig. 1) [2, 3]. Animal breeding provides long-term and cumulative improvement of animal population, serving as the key focus for meeting the growing demands of the world population for sustainable food production. Worldwide there is growing pressure to increase production efficiency and reduce negative environmental consequences of the livestock industry (e.g., greenhouse gas emissions), while maintaining sustainable food production and animal health. The use of genetic and breeding tools has been fundamental to achieving efficient and balanced genetic improvement in livestock populations [4–6].

**Fig. 2:**
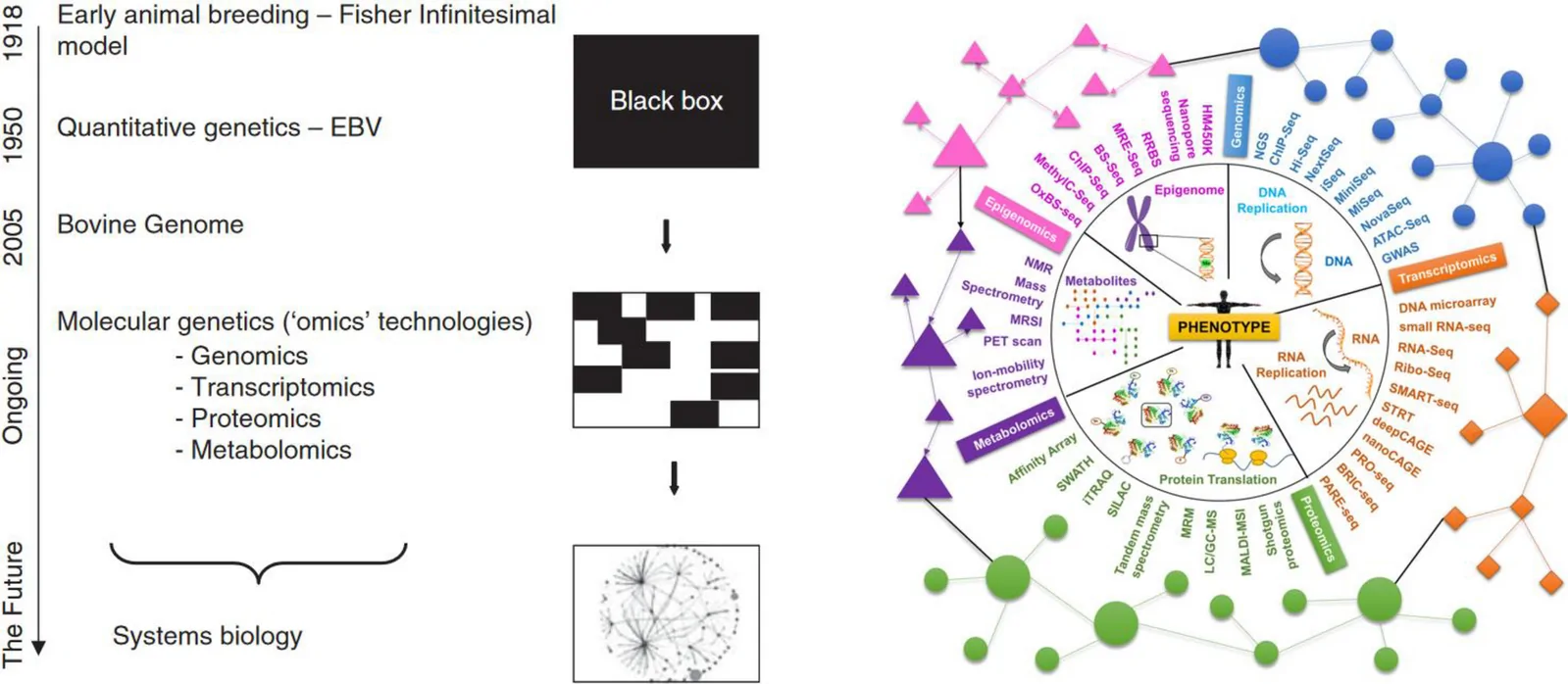
Livestock breeding over time using bovine as an example (left) [8]. Different layers of omics data in human studies (right) [9]

Over the past 100 years livestock breeding has steadily evolved in both theory and application along with new data sources becoming available over time (Fig. 2). The principal steps in livestock breeding include setting up breeding goals, data collection (e.g., phenotyping, genotyping), genetic parameter estimation, understanding genetic architecture of the trait, genetic and genomic evaluation, selection and mating. The livestock breeding communities have developed methods in each of the steps over years and successfully integrated genomic information into selective breeding, and achieved substantial success in combining large-scale phenotypic, pedigree, and genomic information into animal genetic improvement. With the genetic basis of complex traits being better understood day by day, livestock breeding has potential to further evolve from “assuming a black box of genome function” to “integrating biological information into modelling” by deploying inputs from system biology and/or functional genomics (Fig. 2). The scale of omics data (e.g. phenotypic data, genomic data, gene expression information, epigenetic data) has rapidly increased in recent years in human, plant and animal genetics, providing extra functional information for modelling complex traits (Fig. 2). Molecular variation in animal genomes linked to complex phenotypes (e.g. quantitative trait loci, expression quantitative trait loci) are mapped and require state-of-the-art statistical modelling to meet the complex structure of omics data. Alongside established statistical approaches, rapidly developing methods based on artificial intelligence (AI) provide additional opportunities for modelling high-dimensional and potentially nonlinear structures in genomic and omics data [7]. Meanwhile, AI also provides useful tools to develop new or complement methods for phenotyping difficult-to-measure traits, therefore directly benefiting selective breeding.

**Fig. 3:**
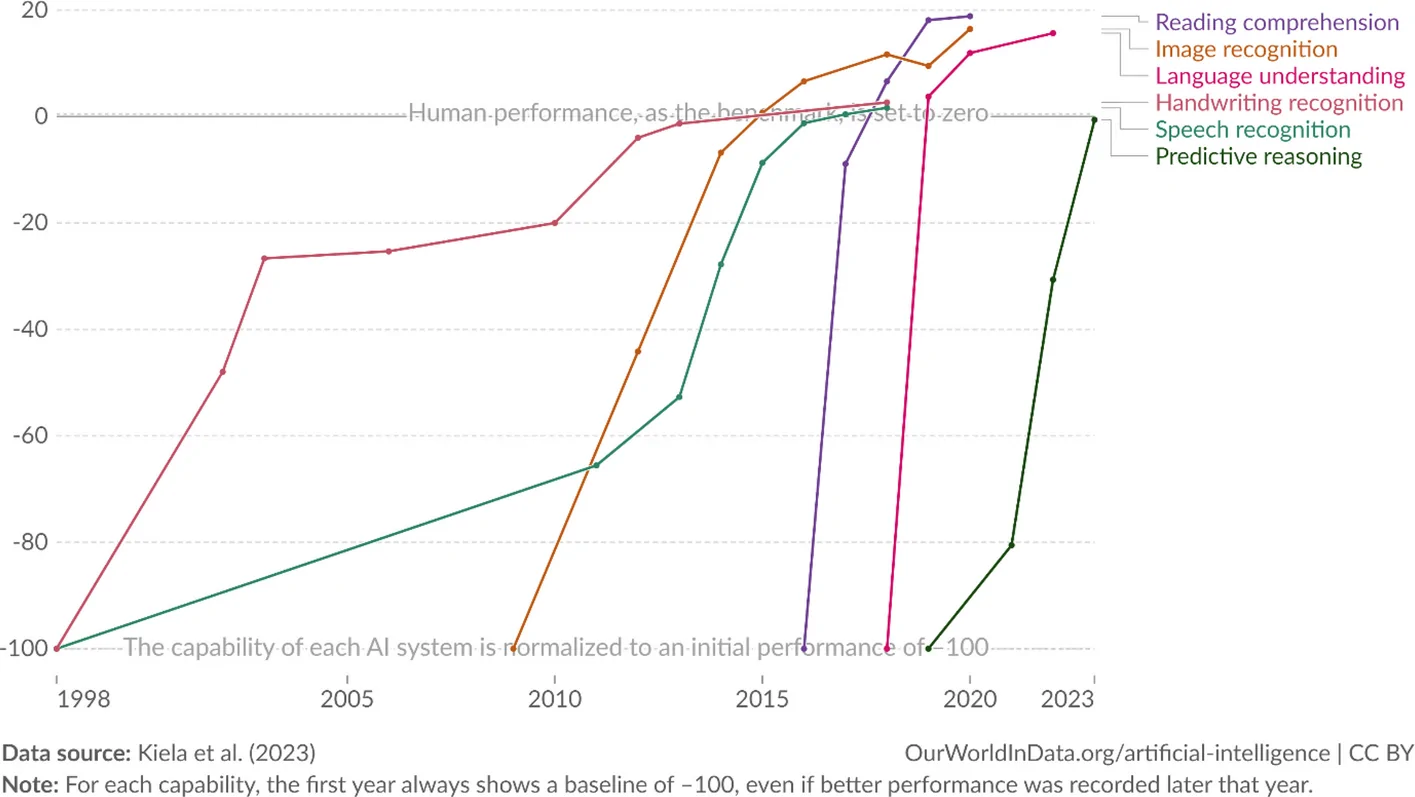
Temporal evolution of test scores of AI systems on various capabilities relative to human performance. The latter is set to zero as the benchmark. The capability of each AI system is normalized to an initial performance of −100 [10]

Artificial intelligence is rapidly expanding into solving multiple complex tasks (Fig. 3). In this review, we use AI as a broad term referring to computational approaches that learn predictive or representational patterns from data, while recognizing that the boundaries between AI, machine learning (ML), and statistical learning are not always strict. Machine learning refers primarily to data-driven algorithms such as random forests, gradient boosting, support vector machines, and related approaches, whereas deep learning (DL), as a subset of ML, refers to multilayer neural-network architectures such as artificial neural networks and convolutional neural networks. Classical quantitative-genetic approaches, including (G)BLUP and Bayesian Alphabet models, are not considered as AI methods in this review, but hybrid frameworks that integrate ML or DL components into these established statistical models are also discussed in this review. This terminology is used throughout the review to facilitate comparison among classical statistical, machine/deep learning, and hybrid approaches in livestock genetics and breeding.

**Fig. 4:**
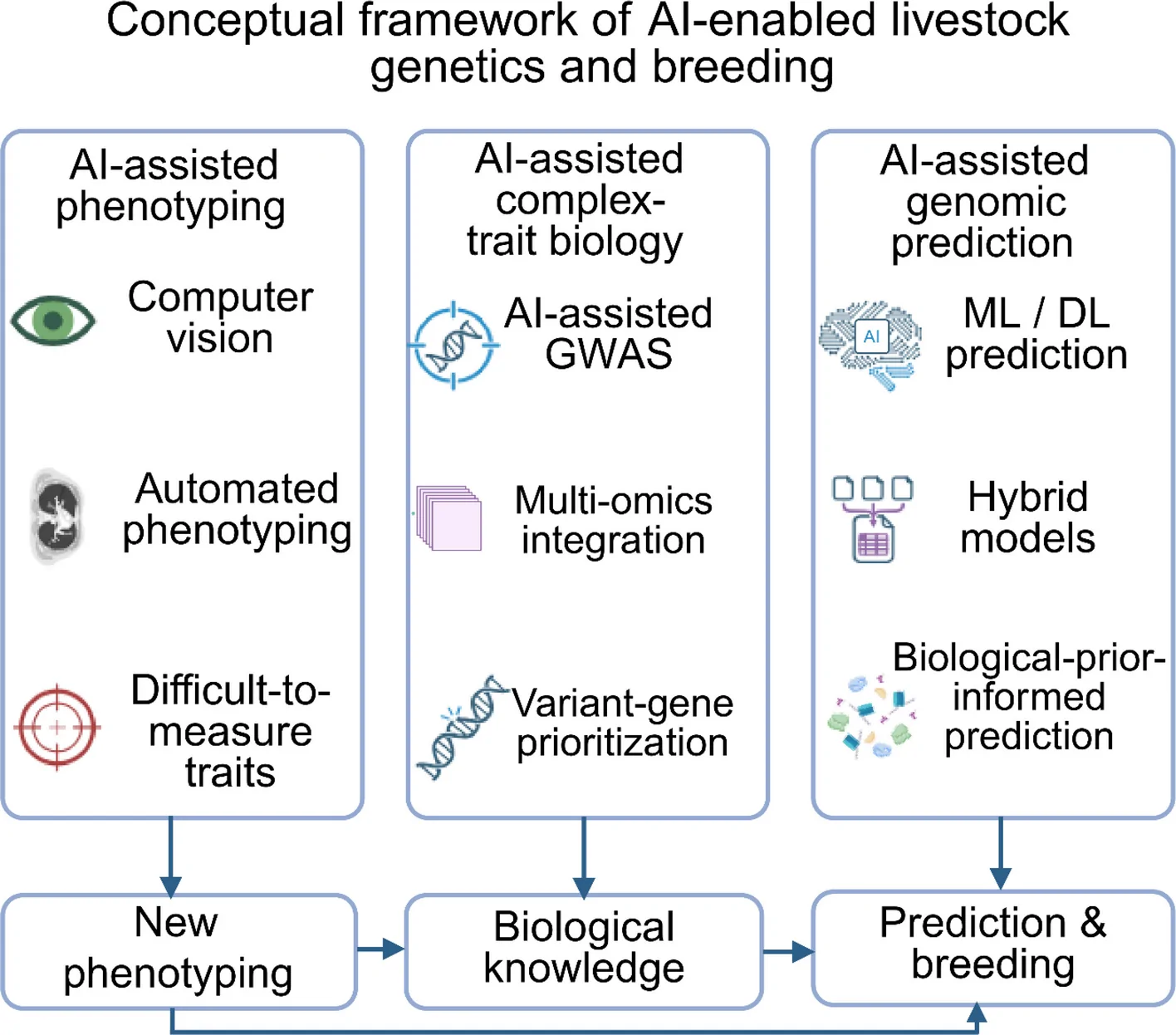
Conceptual framework of AI-enabled livestock genetics and breeding. Artificial intelligence (AI) contributes to livestock genetic improvement through interconnected activities: AI-assisted phenotyping, AI-assisted discovery of complex-trait biology, and AI-assisted genomic prediction. AI-based phenotyping approaches enable scalable and automated measurement of complex traits, directly contributing to genomic analyses and breeding; AI-assisted genome-wide association studies (GWAS) and/or multi-omics integration provide extra tools for prioritization of functional variants associated with complex traits. Machine learning (ML) and deep learning (DL) approaches, together with hybrid models that incorporate classical mixed-model methods or biological information, might provide extra tools to improve genomic prediction in the long run with increasing data size and complexity

Recognizing the potential of AI, this study reviewed the recent developments of AI-based methods in three major areas in livestock genetics and breeding: (1) AI-assisted phenotyping; (2) AI-assisted dissection of complex-trait biology, including AI-assisted GWAS and multi-omics integration; and (3) AI-assisted genomic prediction. These three areas are interconnected, with AI-derived phenotypes providing improved inputs for genetic analyses, AI-assisted genomic and multi-omics analyses generating biological basis and functional priors for complex traits, and these phenotypes and biological insights ultimately contributing to genomic prediction and breeding applications (Fig. 4).

## AI-assisted phenotyping

Accurate measurement of economically important traits on individual animals is fundamental for elucidating their genetic potential and achieving efficient genetic improvement. However, traditional phenotyping approaches, especially when relying on manual or invasive assessments, can be limited by low efficiency, subjectivity, and poor scalability, which hinder their application in large-scale phenotyping. So far, AI-based phenotyping methods have been developed to assist animal identification [11–16]. Accurate animal identification is an important prerequisite for automated phenotyping as it enables longitudinal tracking and integration of repeated phenotype records at the individual level. Conventional electronic identification systems, such as RFID-based approaches, have been used in livestock production in many countries, but they require dedicated hardware and maintenance and may not always support continuous and remote monitoring. AI-based computer-vision approaches can provide a complementary solution by identifying individual animals from visual characteristics such as facial features, body patterns, or other image-derived features, thereby facilitating non-invasive and longitudinal tracking of individual animals and providing an important foundation for continuous and automated phenotyping. Beyond animal identification, AI has been used to derive new phenotypes especially for difficult-to-measure traits (e.g., feed intake). For the established traits already applied in animal breeding, AI-assisted phenotyping has potential to enhance genetic progress by improving efficiency, accuracy, and frequency of phenotyping. So far, AI-based phenotyping methods have been developed to measure a wide variety of traits including morphological phenotypes (e.g., body size and composition) [12, 14, 17–19], behavioral and welfare phenotypes (e.g., activity, movement, and posture) [15, 20–22], and various economically-important phenotypes (e.g., weight and weight gain, feed and energy intake, energy balance, health and disease, fertility) [15, 23–36]. Among an extensive number of studies conducted recently in this area, in this review we illustrate some typical examples to infer the general use of AI in livestock phenotyping.

### AI-assisted estimation of body size, weight, and composition

The use of AI enables non-invasive and automated estimation of body size, weight, and composition for livestock at the individual level—supplementing or potentially replacing labor-intensive methods in future livestock farming. Earlier studies have explored the possibility of predicting body size and weight from morphological features using computer-vision techniques and linear regression models [37–40]. To overcome the limitation of linear regression methods in modelling complex and nonlinear relationships between predictors and phenotypes, machine-learning methods are rapidly evolving for the prediction of livestock morphological features from computer-vision [17–19, 24, 27, 28, 41]. In cattle, Gebreyesus et al. [24] used 3D-camera images of cows plus real body weight measurements to train supervised ML models that predict body weight for individual cows from image-derived features, achieving reasonable prediction accuracies with mean correlation coefficient of 0.94, root mean squared error of 33 kg and a corresponding mean absolute percentage error of 4%. Meanwhile, Stephansen et al. [18] predicted body condition score (BCS) in Jersey cattle using 3D images and ML methods, including gradient boosting machine (GBM) and deep learning using artificial neural network as classification models, where their deep learning model achieved a mean accuracy of 48.1% in predicting the exact BCS phenotype and 93.5% accuracy in predicting BCS with a deviation of 0.5 on a 1–5 BCS scale. In sheep, Robson et al. [17] applied neural networks, in the form of a Generative Adversarial Network, to perform image-to-image processing to predict key ovine phenotypes such as gigot geometry and tissue distribution from sheep CT scans, resulting in predicted values for each image on average 101.44% of the ground truth value with a standard deviation of 12.90% (*n* = 32) across all phenotypes. In swine, camera-based systems have been implemented for foot and leg scoring in pigs, converting categorical human scores into more continuous digital phenotypes and thereby improving the heritability and potential selection response of morphological traits [42]. For pig body weight, Dong et al. [28] introduced a dual-network architecture comprising a Contour Information Extraction Network and a Liveweight Estimation Network, capable of real-time, non-contact estimation of pig body weight at 1,131 frames per second, with an *R*^2^ value of 0.993. In poultry, Sun et al. [19] developed a two-stage CT-based phenotyping framework: A customized segmentation model was first used to identify broiler leg regions from CT images with an intersection over union (IoU) exceeding 95%, after which a random forest regressor was used with the image-derived features to predict broiler leg weight, achieving an *R*^2^ of approximately 0.89. Overall, these examples demonstrate the potential of AI-based models to predict body size, body weight, and body composition of farm animals from computer vision data with high efficiency and accuracy.

### AI-assisted estimation of behavioral phenotypes

Behavioral traits are generally difficult to measure due to labor-intensive and subjective phenotyping strategies. The application of AI to predict behavioral phenotypes, such as activities, movement patterns, and posture, is being investigated in farm animals. In cattle, Ghassemi et al. [21] reviewed pose-estimation approaches that use image and video data to detect and track anatomical key points, thereby enabling quantitative characterization of posture, locomotion, and other behavioral patterns. In swine, image-based phenotyping has also been used to quantify daily eating, drinking, lying, sitting, standing and walking distance in purebred pigs, allowing genetic parameters of difficult-to-measure behavioral traits to be estimated (heritability estimates for studied pig behavior traits ranged from 0.19 to 0.57) and linked to production traits such as average daily gain, backfat thickness and loin depth [43]. Meanwhile, Chen et al. [22] developed a semi-supervised pipeline to automate long-term tracking and monitoring of the activities of group-housed pigs, where the pipeline can be rapidly deployed as only one top-view RGB camera and the average tracking error was reported as 17.99 cm among studied datasets. In sheep, Barwick et al. [44] applied a moving window behavior state classification algorithm to categorize grazing, standing, walking and lying behavior classes using data collected from collar, leg and ear mounted accelerometers on Merino ewes. Accuracy of identifying sheep behavior classes was reported highest for the ear-mounted sensor (86%–95%), followed by the collar-mounted sensor (67%–88%) and leg-mounted sensor (48%–94%) [44].

These studies demonstrated the potential of AI-assisted approaches for more objective and automated behavioral phenotyping in livestock. However, their practical use depends strongly on the cost of the equipment and the potential influence of the equipment on animals and the production environment. Animal-mounted devices, such as collars or accelerometers, can provide detailed individual-level behavioral data, but their large-scale use may be constrained by equipment cost, deployment and maintenance requirements, and potential animal-safety or welfare concerns. Therefore, while such systems are valuable for research and targeted monitoring, further evaluation is needed before their widespread application in commercial populations. Non-contact approaches, including computer-vision-based monitoring, may provide alternative routes for large-scale behavioral phenotyping, although their robustness across individuals, farms, and environments also requires continued examination and validation.

### AI-assisted estimation of other economically important traits

The use of AI has emerged as a promising approach for enhancing the phenotyping of a wide range of economically important traits in farm animals. In dairy cattle, one of the most extensively investigated applications is the use of AI models to infer costly and difficult-to-measure traits in dairy cattle from milk mid-infrared information (MIR) [29–32, 34, 35, 45–69]. Milk sampling of dairy cattle is routinely carried out, and milk MIR spectroscopy is a low-cost method of analysing milk samples. MIR spectroscopy measures the frequencies of infrared light which are absorbed by a sample. These absorption patterns can be thought of as chemical fingerprints of the atoms and structures within a milk sample [70]. Therefore, in addition to the milk composition, MIR spectra can provide information about many other compounds contained within a milk sample [70]. Extensively documented examples include using milk MIR information and AI-based models to predict milk fatty acid composition [30, 34, 50, 55], milk protein composition [64–68], cow health and disease resistance [29, 34, 53, 60, 63], chronic stress [49], feed intake and energy intake [31, 35, 45, 52, 54, 56, 69, 71], body energy balance [31, 35, 53, 54], nitrogen use efficiency [48], reproduction and fertility [32, 53, 61, 62, 72], and environmental traits like methane emission [46, 47, 57–59]. In addition to the utilization of milk information, feed intake of individual dairy cows was also predicted using 3D camera system in large-scale in-house commercial dairy cattle population [25].

In swine, low-cost machine learning pipelines using two-dimensional images have been explored to predict pig growth and carcass traits [73]. Similarly, Wei et al. [74] proposed a deep learning-based system for automated carcass trait determination using images from 3,912 pigs, reporting high accuracy for carcass measurement assessments. Ultrasound images were reported to be used for predicting intramuscular fat in live pigs [75], and used for measuring back-fat depth to determine body composition parameters of fat mass and fat-free mass in growing-finishing pigs [76]. In addition, statistical models based on machine and deep learning were also used as alternatives to linear models to predict growth traits [77], lameness [78], and health status from feeding behaviors [79] in pigs.

In chickens, AI-assisted phenotyping has been increasingly applied to automate and improve the measurement of production and health traits. In broiler, AI-based systems can integrate data from cameras, feeders, load cells, and environmental sensors to monitor feed intake, body weight, activity, health indicators, and environmental conditions in real time [80–83]. Computer vision has also been used for flock-level phenotyping and health monitoring, where Depuru et al. [84] applied real-time AI-based detection and tracking of broiler chickens for health monitoring.

In sheep, recent studies suggested that non-invasive technologies, including video tracking, wearable accelerometers, sensors, thermal cameras, and ultrasound, can be used to predict sheep feed and energy intake [85, 86], lameness status [87], heat stress [88, 89], carcass traits [90], lamb survival [91], and reproductive events [92–94]. Estrus-related phenotypes such as increased locomotion, vocalization, mounting, tail wagging, and interactions with rams can be detected using video tracking, accelerometers, electronic estrus detection systems, or sensors [92–94]. In addition, models based on machine/deep learning were also used for early prediction of adult wool growth and quality [95] and sheep carcass traits [96] using predictor traits in early life.

For future development it will be essential to establish unified phenotyping standards and open data repositories, as well as enhanced interpretability and cross-scenario generalization of AI models. Bhujel et al. [97] systematically reviewed 58 public livestock vision datasets and highlighted that the limited quantity of high-quality annotated datasets collected from diverse environments, animals, and applications, as well as the absence of contextual metadata, are the real bottleneck in precision livestock farming. This underscores that the generation of large-scale, high-quality, diverse, and well-aligned datasets is key to unlocking the full potential of AI-assisted phenotyping, but this will require substantial work in data harmonization, standardization, quality control, and external validation.

## Use of AI to understand complex-trait biology

The scale of omics data has recently expanded dramatically in animal populations driven by advances in high-throughput sequencing. Integrative analyses of multi-omics datasets using statistical modeling and population genetic approaches have enabled the systematic mapping of molecular variants underlying complex traits. In this context, AI methods show potential for modeling high-throughput data with high-dimensional and non-linear structures by capturing intricate relationships among multi-layer information. Recent applications include AI-assisted genome-wide association studies (GWAS) and AI-assisted multi-omics integrative analyses, which complemented and potentially improved the identification of causal variants and refined the understanding of the genetic architecture of complex phenotypes. Accurate identification of variant–trait associations offer valuable insights into complex-trait biology and biological priors for genomic selection and genome editing.

### AI-assisted genome wide association studies

Statistical and computational limitations of genetic analyses have meant that, commonly, primarily quantitative trait loci (QTL) of relatively large effects have been identified, leaving the causal variants and biological mechanisms underlying much of the polygenic variation unresolved [98, 99]. The statistical power of GWAS is influenced by factors including linkage disequilibrium between genotyped markers and causal variants, the effect sizes and allele frequencies of causal variants, sample sizes, and the statistical models employed [100, 101]. Most livestock GWAS have primarily relied on SNP markers, which may incompletely capture other forms of genetic variation (e.g., structural variants, SV), as a recent cattle pangenome study reporting approximately 40% of SV not tagged by nearby SNPs based on linkage disequilibrium [102]. In addition, conventional GWAS analyses could suffer from a lack of power and accuracy when dealing with the complexity of multiple interactions or correlations between predictors (e.g., SNP–SNP and SNP–covariate interactions) [103–107]. In such a context, with rapid expansion in data scale and computational resources, new opportunities including AI methods might provide new strategies to uncover hidden patterns in the associations between genome and complex traits, serving as alternative or complement to classical GWAS methods in different data contexts.

The potential benefits of using AI to improve GWAS have been explored using simulated datasets [108, 109]. Minozzi et al. [108] used simulated genomic data and phenotypic data of yield deviations on 3,000 individuals to perform GWAS using Random Forest (RF) and GRAMMAR-CG (Genome wide Association using Mixed Model and Regression—Genomic Control), where both methods were successful in identifying the largest QTL for yield deviations. Zhao et al. [109] developed a NN-Bayes method that incorporates Bayesian Alphabet models into non-linear neural networks via hidden layers between SNPs and observed traits to perform GWAS using simulated data as well as real data for pig backfat. In simulated dataset the study observed better model performance of NN-BayesC*π*−2 (2 hidden nodes with priors in NN-BayesC*π*) compared with LM-BayesC*π* (a linear model with BayesC*π* prior) for GWAS. In real pig data, the GWAS results from NN-BayesC*π*−2 showed a similar pattern and prioritized the same genomic window that was identified as significant by the linear mixed-model association analysis using the same data [109]. Generally, studies using simulated data indicated the effectiveness of AI-based models in association analyses.

Despite the potential benefit shown from simulated data, the performances of AI-assisted GWAS were still uncertain when using real data. So far AI tools have been applied to a few genome-wide association analyses in livestock species (e.g., cattle, pig, sheep, chicken) for SNP ranking, marker feature selection, and prescreening candidate genes for complex traits:

In cattle, Yao et al.[110] applied Random Forest-based GWAS to identify additive and epistatic SNPs associated with residual feed intake (RFI) in Holstein dairy cattle. The SNPs were ranked using importance scores of all SNPs generated by RF considering both main effects of variables and interactions between variables, resulting in 188 top-ranked SNPs based on the RF importance threshold, among which 38 SNPs were mapped to QTL regions for RFI reported previously in beef cattle. Additionally, 25 most frequent pairwise SNP interactions were reported as possible epistatic interactions for RFI by analyzing the tree structures produced within RF [110]. Similarly in beef cattle, RF-based GWAS for age at first calving (AFC) in Nellore cattle ranked SNPs by the importance score of each SNP across independent RF analyses according to their predictive relevance, identifying candidate genes for AFC related to cattle fertility that agrees with previous literature [111]. They also revealed that some top-ranked SNPs from the RF-based GWAS did not present a strong marginal linear effect but were potentially involved in epistatic hotspots between genomic regions on different autosomes for AFC [111]. A similar study on Canchim beef cattle used RF to associate SNPs with de-regressed breeding values for backfat thickness, followed by regression analyses to estimate the amount of variance explained by the top SNP selected from RF analyses [112]. Apart from RF, other ML methods including GBM and Extreme Gradient Boosting (XGBoost) had also been adopted in genome-wide scans to select for top SNP for live weight of beef cattle for genomic prediction [113]. In addition, a few studies were carried out to compare RF-based GWAS with classical GWAS approach in cattle. Li et al. [114] performed GWAS for pregnancy status of Santa Gertrudis cows using RF method as well as Logistic Regression (LR), resulting in two SNPs in common among the top 10 SNPs from the two methods but the rest SNP association profiles being significantly different. A literature search revealed that candidate genes identified by the RF analyses were more previously found associated with fertility traits, whereas none of the candidate genes identified by LR seemed to have known effects on reproductive performance. Recently, Lai et al. [115] performed GWAS for foot lesion phenotypes in Holstein cattle using three GWAS approaches including generalized linear mixed model (GLMM) regression, chunk-based association testing (CBAT), and RF approach. The GLMM and CBAT approaches revealed the same candidate genomic regions from associations, but not from the RF approach. It was reported that the RF models tended to be overfitted and showed poor phenotype prediction in this study for foot lesion phenotypes, so that the SNPs exceeding the predefined RF importance threshold were considered likely false-positive candidates in this study [115].

In pigs, GWAS using GBM and rrBLUP [116] were compared with a classical single-marker association analysis implemented in PLINK [117] to identify markers associated with congenital anomalies and piglet splay leg, where they found five SNPs were commonly prioritized by all three approaches and an extra group of markers detected by each two methods [118]. They also mentioned in this study that GBM was more accurate than classical single-marker association in labeling the affected piglets as affected, but at the cost of a higher false-positive rate [118].

In poultry, RF-based association was used to identify SNPs associated with chicken eggshell strength, where RF algorithm was used to estimate the relative importance of each SNP regarding prediction of de-regressed breeding values for the trait, followed by downstream multi-omics integration to further prioritize candidate genes from association [119]. Similar RF-based association was also applied to identify informative SNPs for bone stability traits (bone breaking strength and bone mineral density of the tibiotarsus and the humerus) in laying hens [120].

Extending the concept to sheep, a recent study used a two-stage ML-enhanced GWAS to identify biologically important markers for wool traits in Central Anatolian Merino Sheep [121]: In the first stage, they conducted feature selection (using LASSO, Ridge Regression, and Elastic Net) to generate a consensus SNP panel per trait; In the second stage, association analyses were conducted using RF, XGBoost, and Support Vector Regression (SVR) to prioritize SNP from the most predictive models (*R*^2^ up to 0.86). After the two stages the identified candidate genes were biologically relevant loci for the studied traits, supporting the utility of ML-enhanced GWAS pipelines in identifying biologically important markers in this case. In sheep, to compare RF-based GWAS with classical GWAS approach, Li et al. [114] performed GWAS for sheep coat pigmentation by single SNP association and RF approaches, where both methods were able to identify the largest-effect markers on Chromosome 14 being the most strongly associated with sheep coat pigmentation despite the rest of the signals significantly different.

Overall, based on the studies so far, AI methods have shown effectiveness of modeling complex genetic architectures with potential benefit in better modeling the residual polygenic and non-additive genetic effects. The simulated data have indicated potential benefits of combining AI into association analyses (e.g., epistatic effect between markers), where this potential benefit was shown in a few studies [110, 111]. These potential benefits still require systematic validation in real datasets, particularly through rigorous benchmarking against classical GWAS, appropriate control of population structure and relatedness, independent-population validation, and careful assessment of false-positive rates. Future AI-based SNP prioritization may serve as an extra useful tool for causal variant identification rather than replacing classical association testing, and the benefit of AI-based methods might become larger when data sizes/types increase and technologies evolve. Currently, machine-learning applications in livestock association analyses are dominated by relatively established and interpretable approaches, particularly tree-based ensemble models (e.g., RF, GBM, and XGBoost) and kernel-based methods (e.g., SVM and SVR), which are commonly used for SNP-importance ranking, feature screening, and construction of reduced candidate-marker sets. In contrast, direct applications of deep-learning and generative-AI approaches to livestock GWAS remain relatively limited. This is partly due to the comparatively small sample sizes of livestock datasets relative to the high dimensionality of genome-wide markers, which can restrict the effective training and benchmarking of more parameter-rich neural-network models. Therefore, the studies summarized above primarily reflect the methods that have so far been practically evaluated in livestock populations rather than the full methodological scope of modern AI. In contrast, the integration of AI into GWAS has been more broadly explored in human genetics, where various methods and frameworks have been investigated and might be transferred, adjusted, or combined into future AI-assisted association in livestock species. For this purpose, we will review some significant human studies/applications on AI-assisted GWAS in the Future perspectives of this review.

### Integrative multi-omics studies by AI

The majority of trait-associated variants identified by GWAS, commonly estimated at approximately 90% in human, reside in non-coding regions [122–124]. These functional variants in non-coding regions exert their effects through the regulation of molecular phenotypes such as gene expression, chromatin accessibility, or DNA methylation. These molecular phenotypes, derived from multi-omics data (e.g., transcriptomics, epigenomics, chromatin accessibility profiles), represent the regulatory cascades that connect DNA sequence variation to cellular/organismal traits, where the variations of molecular phenotypes are also partly heritable from generation to generation.

The systematic characterization of regulatory effects of genomic variants is essential for inferring molecular mechanisms underlying complex traits, contributing to selective breeding and genome editing. Recent multi-omics research integrated large-scale GWAS and multi-omics data to decipher regulatory variations underlying complex traits in cattle, pig, chicken, and sheep for economically important traits including production, health, fertility, efficiency, and conformation [125–135]. Substantial breakthroughs have been achieved in recent years particularly through large collaborative initiatives: The Farm Animal Genotype–Tissue Expression (FarmGTEx) Project [125] has released open-access multi-tissue regulatory resources for cattle, pig, sheep, and chicken, establishing transcriptome-wide expression QTL (eQTL) and splicing QTL (sQTL) atlases across 30–50 tissues within each farm animal species; The Functional Annotation of Animal Genomes (FAANG) consortium [136, 137] produced high-quality chromatin accessibility, histone modification, and DNA methylation maps for key farm animal species, laying essential foundations for model training and regulatory inference in livestock species.

Deploying solid resource construction, AI-based multi-omics integration has begun to be applied to farm animals. For example, Xiang et al. [138] developed a novel Genetic Score Omics Regression framework in cattle, extending transcriptome-wide association studies (TWAS) to large cattle reference populations to detect cis-regulatory effects shaping bovine complex traits. In pig, a general convolutional neural network (CNN) framework for multi-omics integration has been applied, achieving balanced performance for candidate-gene prioritization (precision 72.9%, recall 73.5%, F1 73.4%) [139]. In sheep, a network-based ML pipeline combined weighted gene co-expression networks from milk somatic cell transcriptomes with gene–term networks was trained on latent gene-importance representations, and then integrated these embeddings with gene-level pleiotropy P-values in a hierarchical model to prioritize pleiotropic candidate genes linked to milk production/quality and health-related processes [140]. In summary, AI-based approaches have been increasingly adopted for integrating animal omics data and have shown promising performances in predicting functional variants for livestock complex traits.

Despite these advances, AI-based multi-omics integration in livestock faces several challenges, including relatively small sample sizes compared with the high dimensionality of molecular features, heterogeneity across studies and experimental platforms, incomplete matching of omics layers across individuals, and context-specific regulatory effects across tissues, developmental stages, and environments. Moreover, high predictive performance does not necessarily imply causal relationships between genomic variation, molecular phenotypes, and complex traits. Future studies should therefore emphasize external validation across independent populations, breeds, tissues, and environments, careful handling of biological and technical heterogeneity, and integration with established statistical-genetic and causal-inference approaches. As livestock multi-omics resources continue to expand, AI is expected to play an increasingly important role in integrative analyses and mechanistic dissection of complex traits, while emerging Transformer-based and other sequence-learning models may further facilitate the transition from association-based analyses toward sequence-to-function prediction.

## AI applied to genomic prediction

Classical genomic prediction models, primarily the genomic best linear unbiased prediction (GBLUP, single-step GBLUP) and the Bayesian alphabet family, have been well-established as the foundation of genomic evaluation in livestock breeding [141, 142]. With the rapid advancement of AI, a growing number of nonlinear approaches based on machine and deep learning algorithms emerged in animal breeding to investigate its potential in improving prediction performances through capturing nonlinear interactions, non-additive effects, and genotype-by-environment (G × E) effects that potentially shape complex traits.

Rather than replacing classical models outright, several studies have sought to enhance classical GBLUP and Bayesian frameworks by integrating machine learning components, achieving improved predictive performance while retaining the interpretability and stability of mixed-model approaches. Zhao et al. [109] developed NN-Bayes, a Bayesian neural network that embeds Bayesian Alphabet prior within a nonlinear architecture to capture dominance and epistatic signals while retaining interpretability. In simulation studies, NN-Bayes outperformed conventional linear Bayesian models in 94.9% of validation tests and showed significant accuracy gains in 16 out of 20 simulated traits, although real-data improvements tended to be marginal. Building on this work, the authors further expanded their framework to model intermediate omics layers and nonlinear regulatory cascades by introducing NN-MM, a neural-network generalization of BLUP, which achieved markedly higher prediction accuracy than the single-step approach under high omics missingness (> 80%) and consistently outperformed linear models when the underlying omics–phenotype relationship was nonlinear [143]. Similarly, Shokor et al. [144] developed DLGBLUP, a hybrid framework integrating deep learning with GBLUP to capture nonlinear inter-trait relationships in livestock. From simulation data DLGBLUP was shown consistently more accurate predicted genetic values for traits simulated with strong nonlinear genetic relationships. However, when applied to real data from the French Holstein dairy cattle population, DLGBLUP detected nonlinear genetic relationships between traits but no significant increase in prediction accuracy was observed [144]. In addition, Yin et al.[145] developed KAML, a kinship-adjusted machine-learning method that optimizes pseudo-QTNs and SNP weights via cross-validated marker selection to improve genomic prediction for complex traits tested in both simulated datasets and real data including human, cattle, horse, and maize datasets. In their results KAML outperforms linear mixed models in prediction accuracies in most studied traits and produced comparable prediction accuracy to Bayesian methods but with faster computation time compared to Bayesian methods [145].

Aside from hybrid strategies that combine deep learning with classical mixed models, recent work has explored the direct use of AI methods to model the relationship between genomic markers and complex traits, where the prediction relies entirely on nonlinear learning. However, based on reported results so far, the improvement in prediction performances is marginal when using pure AI models compared to classical models of GBLUP and ssGBLUP. Lourenco et al. [146] comparatively evaluated the genomic predictive performance of regularized regression methods, deep, ensemble and instance-based learning models using one simulated animal breeding dataset and three empirical maize breeding datasets, where in this study they reported that no single AI model consistently outperformed others or surpassed classical mixed-model approaches across traits. Similarly, Xiang et al. [147] compared GBLUP with multiple AI-based models (CNN, SVM, XGBoost, RF) for genomic prediction on productive traits in pigs, where the GBLUP model achieved comparable prediction accuracies with the best-performed machine learning model for almost all the studied traits. In contrast, some studies reported small improvement in prediction performances when certain machine learning algorithms were applied to certain traits in pigs, but the general improvement was small and not significant in most cases when considering standard errors [148, 149]. In cattle, similar results were reported for genomic prediction on behavior traits in dairy cattle, where deep learning methods improved prediction accuracy for behavior traits on a marginal scale with no significant statistical difference compared to GBLUP [150]. In poultry, Liu et al. [151] systematically compared seven ML algorithms with GBLUP and Bayesian methods to predict the genomic breeding values of laying traits, growth and carcass traits in a yellow-feathered broiler breeding population. Their results indicated that classic methods, such as GBLUP and Bayesian, achieved better or comparable prediction accuracy compared to ML methods in seven of the eight traits studied, but ML methods showed a significant improvement in accuracy over GBLUP and Bayesian methods for one trait of half-eviscerated weight in the studied chicken population [151].

It is noted that the prediction performance of AI-based models is influenced by trait genetic architecture, feature selection, AI method used, and hyperparameter optimization [152]. Studies consistently show that artificial neural networks (ANN) and several nonlinear ML models (such as Multivariate Adaptive Regression Splines, RF, Bagging, and Boosting) excel when traits are influenced by epistasis or other nonlinear effects. In contrast, GBLUP often achieves the highest accuracy for highly polygenic traits, underscoring the continued competitiveness of linear models in additive, infinitesimal genetic architectures [153]. The majority of nonlinear AI methods require careful hyperparameter tuning, and their performance can decline rapidly without adequate optimization. Artificial neural networks are particularly sensitive: when the number of markers greatly exceeds the number of training samples, feature selection and strategies such as seeded initial weights are essential for stable convergence and competitive accuracy. Without these steps, ANN often underperform relative to simpler models [154].

In general, despite the ongoing debate and the clear dependence of AI performance on data structure and trait architecture, AI-based approaches have opened new avenues for genomic analyses, but the methods and contexts in which AI can be integrated with statistical modeling for genetic improvement remain to be explored. Based on findings so far, rather than replacing classical models, integrating ML components into classical GBLUP and Bayesian frameworks might be promising for some traits, potentially improving predictive performance while retaining the interpretability and stability of mixed-model approaches. Looking ahead, with genomic and phenotypic resources expanding, and large-scale multi-omics datasets accumulating, AI methods might play an increasingly important role considering the ability of AI methods to capture high-dimensional nonlinear relationships and integrate diverse biological information into prediction. 

## Future perspectives

Looking forward, future studies are needed to explore the ways for AI to contribute to mating decisions, inbreeding control and maintaining genetic diversity. Several key challenges will determine whether AI can be effectively translated into accurate and practical tools for livestock genetic improvement. First, data standardization and computational resources are foundational: harmonized metadata, benchmark datasets, and transparent evaluation protocols are required to prevent brittle models that fail to generalize, while scalable computational resources and efficient training/inference pipelines are essential for routine deployment. Second, method development and validation are the key to AI applications. Validation between methods and between populations serve as essential steps for reducing false positives and method selection. Third, method development is expected to move beyond isolated AI applications toward domain-specific, multivariate, and multi-model frameworks. Meaningful applications to livestock genetic improvement warrant connecting relevant AI functions (e.g., phenotyping, biological discovery, prediction) into a coherent pipeline: AI-derived phenotypes increase power and reduce noise; AI-integrated genetic analyses yield mechanistic priors (functional variants, genes); and AI-assisted prediction incorporates priors to enhance prediction accuracy and interpretability. More AI applications on mating decisions and inbreeding control can be aligned as well.

In addition, some examples from human genetics might be useful for adapting analogous methodologies to livestock species. In human genetics, the integration of AI into genomic analyses has been pursued broadly and deeper, supported by large-scale biobanks, standardized phenotyping, and extensive functional annotation resources. For example, Li et al. [155] developed DeepGWAS, a deep NN framework that integrates GWAS summary statistics with linkage disequilibrium information and related functional annotations to computationally enhance association signals without increasing sample size for neuropsychiatric disorders. Another representative framework, COMBI [156], combines machine learning with statistical hypothesis testing to identify functionally relevant variants from high-dimensional genomic data. The same research group later introduced DeepCOMBI [157], a deep-learning extension of COMBI designed to uncover SNP associated with traits of interest through end-to-end neural modeling. DeepCOMBI constructs deep neural networks to predict phenotypes directly from genotypes, followed by layer-wise relevance propagation to quantify the contribution of each SNP to the network’s output. In addition, GWANN [158] transformed genomic data into image-like matrices, enabling convolutional neural networks to classify trait-associated variants based on learned spatial patterns of allelic combinations. Although its performance was sensitive to population structure and minor allele frequency distributions, GWANN demonstrated the feasibility of computer vision–inspired strategies in genome analyses. As to AI-assisted multi-omics, the DeepSEA [159] and Sei [160] models represented the fully sequence-based approaches for predicting the functional impact of noncoding variants, by training a deep convolutional neural network on large-scale chromatin profiling data.

Looking ahead, several emerging AI approaches may extend current livestock genetic analyses, although most remain at an exploratory stage. Graph-based models could integrate regulatory networks and multi-omics information for gene and variant prioritization, while causal-learning approaches may help distinguish predictive associations from putative biological mechanisms. Uncertainty-aware learning could provide confidence estimates for AI-derived phenotypes and genomic predictions, particularly when models are transferred across populations or environments. Large language models may facilitate retrieval, synthesis, and integration of heterogeneous biological knowledge and annotation resources, whereas Transformer-based sequence models could support regulatory variant prediction and cross-species functional annotation directly from genomic sequence. The practical values of these approaches remain exploratory in livestock and will require systematic benchmarking, independent validation, and comparison with established statistical and quantitative-genetic approaches.

## Conclusion

Although still in the early stage, AI demonstrates strong potential for broad applicability in animal genetics and breeding. For livestock species, AI-assisted phenotyping offers scalable, non-invasive, and automatic measurements for phenotypes, which directly boosts reference populations and enhances genetic analyses down the line. AI-generated information can also support livestock management through health, disease, and welfare monitoring, to complement long-term animal genetic improvement. At the same time, AI approaches for understanding complex-trait biology, especially AI-assisted GWAS and omics applications, are starting to work alongside classical linear methods to generate biological priors for complex traits by capturing nonlinear, interactive, and context-sensitive patterns. Overall, these AI-generated inputs (from phenotyping and biological priors) are explored for possibilities in improving livestock genetic improvement through classical methods or AI-integrated statistical modeling. Future breakthroughs are likely to be driven by increased data size/type, enhanced computational capacity, standardized data infrastructure, benchmarking between methods, and multidisciplinary collaboration among broader areas in science and technology.

## Data Availability

No datasets were generated or analysed during the current study.

## Abbreviations

AFC: Age at first calving
AI: Artificial intelligence
ANN: Artificial neural network
BCS: Body condition score
BLUP: Best linear unbiased prediction
CNN: Convolutional neural network
COMBI: Compressive combined association test
DL: Deep learning
eQTL: Expression quantitative trait loci
FAANG: Functional Annotation of Animal Genomes
FarmGTEx: Farm Animal Genotype–Tissue Expression
GBLUP: Genomic best linear unbiased prediction
GBM: Gradient boosting machine
GLMM: Generalized linear mixed model
GWAS: Genome-wide association studies
LM: Linear model
LR: Logistic regression
ML: Machine learning
NN: Neural network
QTL: Quantitative trait loci
QTN: Quantitative trait nucleotide
RF: Random forest
RFI: Residual feed intake
SNP: Single nucleotide polymorphism
sQTL: Splicing quantitative trait loci
SVM: Support vector machine
SVR: Support vector regression
TWAS: Transcriptome-wide association studies
XGBoost: Extreme gradient boosting
